# Assessing Auditory Processing Deficits in Tinnitus and Hearing Impaired Patients with the Auditory Behavior Questionnaire

**DOI:** 10.3389/fnins.2017.00187

**Published:** 2017-04-06

**Authors:** Isabel Diges, Francisco Simón, Pedro Cobo

**Affiliations:** ^1^ACURE-Tinnitus and Hyperacusis ClinicMadrid, Spain; ^2^Institute of Physical and Information Technologies, Consejo Superior de Investigaciones Científicas (CSIC)Madrid, Spain

**Keywords:** auditory processing disorder, tinnitus, hearing loss, questionnaires

## Abstract

**Background and Purpose:** Auditory processing disorders (APD), tinnitus and hearing loss (HL) are typical issues reported by patients in audiologic clinics. These auditory impairments can be concomitant or mutually excluding. APD are not necessarily accompanied by significant HL, whereas many adults exhibit peripheral HL and typical cognitive deficits often associated with APD. Since HL, tinnitus and APD affects to several parts of the ascending auditory pathway from the periphery to the auditory cortex, there could be some interrelationship between them. For instance, tinnitus has been reported to degrade the auditory localization capacity. Tinnitus is believed to be triggered by deafferentation of normal peripheral input to the central auditory system. This peripheral deficit can be accompanied by HL or not, since a type of permanent cochlear damage (thus deafferentation) without an elevation of hearing thresholds might persist. Therefore, a combined study of APD, tinnitus and HL on the same cohort of patients can be audiologically relevant and worthy.

**Methods:** Statistical analysis is applied to a cohort of 305 patients attending an audiology clinic in Madrid (Spain). This group of patients is first categorized in four subgroups, namely, HLTG (with tinnitus and HL), NHLTG (with tinnitus and without HL), HLNTG (with HL but no tinnitus), and NHLNTG (neither tinnitus nor HL). The statistical variables include Age, Average Auditory Threshold (ATT), for assessing HL, Tinnitus Handicap Inventory (THI), for measuring tinnitus, and a new 25-item Auditory Behavior Questionnaire (ABQ), for scoring APD. Factor analysis is applied to arrange these items into 4 subscales. The internal consistency reliability of this ABQ is confirmed by calculating Cronbach's coefficients α. The test-retest reliability is assessed by the intraclass correlation coefficients, *ICC*. Statistical techniques applied to the data set include descriptive analysis of variables and Spearman rank correlations (ρ) between them.

**Results:** Overall reliability of ABQ is confirmed by an α value of 0.89 and by an *ICC* of 0.91. Regarding the internal consistency reliability, the four subscales prove a fairly good consistency with α coefficients above 0.7. Average values of statistical variables show significantly lower age of patients with tinnitus and no HL, which can provide a cue of noise overexposure of this segment of population. These younger patients show also decreased ABQ and similar THI in comparison with patients in the other subgroups. A strong correlation (ρ = 0.63) was found between AAT and Age for the HLNTG subgroup. For the HLTG subgroup, a moderate correlation (ρ = 0.44) was found between ABQ and THI.

**Conclusion:** The utilized questionnaire (ABQ), together with AAT and THI, can help to study comorbid hearing impairments in patients regularly attending an audiological clinic.

## Introduction

The auditory system transmits sounds from the environment to the auditory cortex where they are processed to produce a perception. The sound signal, a vibroacoustic wave, is transduced into an electrical train of pulses at the synapses between the hair cells of the Corti organ and the auditory nerve. This interface is a powerful device able to transmit signals from the periphery to the auditory pathway spanning 12 decades in amplitude (120 dB) and 3 decades in frequency (20–20 kHz) (Knipper et al., 2013). This mechano-electrical transduction of sound waves into a train of electrical spikes is completed within 1–4 ms with standard deviation of roughly 0.8 ms, which is even lesser than the corresponding constant time of mammalian visual cells (Kopp-Scheinpflug and Tempel, 2015). Auditory signals are thus reliably transmitted along large diameter axons and across highly specialized synapses through the afferent auditory pathway.

Acoustic signals (coded as electrical spike trains) spread from the auditory nerve to the higher central auditory system through the brainstem, an intricate network of neurons with soma grouped into the ascending auditory nuclei. The auditory brainstem involves many sophisticated auditory processing including frequency analysis, sound localization, temporal integration and discrimination, binaural cues for spatial analysis, multisensorial processing, and others. Processing mechanisms for higher order patterns of sound are carried out in the primary auditory cortex (Griffiths, 2002). Any abnormal deviation of this rather sophisticated sound transduction, coding, and processing system arises auditory impairments, including hearing loss (HL), tinnitus and auditory processing disorders (APD).

Peripheral deficits afford HL or hypoacusis. The prevalence of HL has, due to aging of the population, doubled over the past 30 years (Knipper et al., 2013). HL is usually measured as an elevation of hearing thresholds expressed in dB. However, recent studies have revealed a type of permanent cochlear damage, without an elevation of hearing thresholds (Weisz et al., 2006; Schaette and McAlpine, 2011). This subtle damage should be linked to a permanent and progressive degeneration of auditory fibers that occurs in association with damage of the inner hair cell synapse (Knipper et al., 2013).

Tinnitus is the medical term for the auditory perception of sounds in the absence of any external source. As an auditory phantom perception, it seems to be the correlate of maladaptive attempts of the brain to reorganize due to distorted sensory input (Kleinjung et al., 2009). This notion is confirmed by the finding that HL is the most important risk factor for developing tinnitus and that most people with sudden unilateral deafness experience tinnitus. In general, there is increasing evidence that tinnitus is related to alterations of neuronal functioning in the central auditory system which compensates for diminished input by upregulating its responsiveness in sub-cortical and cortical networks (Eggermont, 2012). This auditory percept presents in a great variety: a rustling, whistling, ringing, murmuring or humming sound (neural sounds) which can come in high or low tones, be loud or soft and be continuous or interrupted. Tinnitus is an uncomfortable symptom affecting severely the quality of life of adults (Holm et al., 2005). This sound sensation may cause many audiological, cognitive and neurological issues ranging from hearing and attention deficits to anxiety, annoyance, irritability, disturbed sleep patterns, and depression (Zeng et al., 2011; Zhang, 2013). Tinnitus as such is not an abnormal sensation. Most people will experience tinnitus after a couple of minutes in a silent anechoic room. Tinnitus that occurs every day for more than 5 min is reported by 10–15% of the population, and for 1–2% it affects their quality of life considerably (Van de Heyning et al., 2007; Hall et al., 2015).

APD refers to difficulties in the auditory mechanisms underlying the following abilities of the auditory system: sound localization and lateralization, auditory discrimination, auditory pattern recognition, temporal aspect of audition, and auditory performance in competing, or with degraded, acoustic signals (ASHA-American Speech-Language-Hearing Association, 2005). Therefore, it is a disorder associated with the impaired capability of the auditory system to process complex sound signals, especially in degraded or noisy scenarios (Griffiths, 2002). It may be associated with difficulties in listening, speech understanding, language development, and learning (Jerger and Musiek, 2000). The prevalence of APD is 2–7% for school-aged children (with a ratio of 2:1 in boys with respect to girls), and 10–20% in the elderly (Skarzynski et al., 2015). APD is not necessarily accompanied by a significant increase in the pure tone hearing thresholds, especially in young people. Furthermore, increased audiometric thresholds cannot fully account for the difficulty that elderly listeners experience in processing speech in noise (George et al., 2007). These concomitant problems make the diagnosis of APD more difficult. In normal hearing patients, APD can be diagnosed using screening tests including competing words, competing sentences, dichotic listening, speech understanding in noise, filtered speech, and phonemic synthesis (Skarzynski et al., 2015; Weihing et al., 2015). More recently, speech-evoked auditory brainstem responses (Kopp-Scheinpflug and Tempel, 2015; Rocha-Muniz et al., 2016) and mismatch negativity (MMN) (Rocha-Muniz et al., 2015) have been proposed to objectively detect APD.

Since HL, tinnitus and APD are associated with pathologies (or alterations) at different locations on the ascending auditory pathway, it can be hypothesized that these might be interrelated. The main goal of this work is to present a correlational analysis to assess associations between HL, tinnitus and APD in a sample of patients exhibiting these hearing disorders. Furthermore, a new measure for assessing APD, the Auditory Behavior Questionnaire is introduced.

## Materials and methods

### Participants

The study sample consists of a cohort of 305 patients, 174 men (age = 44 ± 14 years) and 131 women (47 ± 14 years), attending an audiologic clinic in Madrid (Spain) between 2011 and 2014.

Firstly we performed *post-hoc* comparisons in the basis of HL obtaining two groups: NHLG composed of subjects with normal hearing, and HLG formed with subjects with hearing loss. The threshold for being included in these groups was calculated as the average of the tonal audiometric thresholds (AAT) for 0.25, 0.5, 1, 2, 4, and 8 kHz frequency bands (George et al., 2007; Savastano, 2008):

(1) $$\begin{array}{l} AAT=\frac{1}{6}\overset{6}{\underset{n=1}{\sum }}HL\left(f_{n}\right) \end{array}$$

where *f*_*n*_ are the octave band frequencies between 250 Hz and 8 kHz. Individuals with average threshold under (over) 25 dB were included in the NHLG (HLG). According with this criterion, 195 patients were found to fit into the HLG and 110 into the NHLG.

Then, subjects were classified as suffering of tinnitus (182 patients) or not (123 patients). All tinnitus sufferers were asked to fill in the THI questionnaire (Spanish version proposed and validated by Herraiz et al., 2001). From the 182 tinnitus sufferers, 53 belonged to the NHLG and the other 129 were included in the HLG. From the 123 non tinnitus patients, 57 belonged to the NHLG and the other 66 were included in the HLG. The categorizing of patients is summarized in Table 1.

**Table 1:** **Categorizing of patients**.

**Normal Hearing Group (NHLG)**		**Hearing Loss Group (HLG)**	
110		195	
**Tinnitus (NHLTG)**	**Non tinnitus (NHLNTG)**	**Tinnitus (HLTG)**	**Non tinnitus (HLNTG)**
53	57	129	66

### Measures

All patients referring hypoacusis, tinnitus, hyperacusis, APD, acoustic distortion, aural pressure, acoustic trauma, or otalgy were selected for this study. All of them were subjected to audiological and ENT explorations. Audiological exploration was carried out into an audiological cabin, using a two-channel clinic audiometer AC40 from Interacoustics, including tonal audiometry, logoaudiometry, discomfort threshold, and distortion products otoacoustic emissions (DPOAEs). ENT exploration consisted of otoscopy, rhinoscopy, and faringoscopy. Besides AAT and THI, all patients were asked to fill in the Auditory Behavior Questionnaire described in the following Section.

### The auditory behavior questionnaire

A large number of validated questionnaires have been proposed for quantifying tinnitus distress, disability or handicap. The patient responses to these questionnaires are summed resulting in a final score, which is then used to rate their tinnitus severity. A percentage of tinnitus patients also report listening difficulties typically related with the auditory processing disorder. Although, some of these questionnaires include auditory perceptual difficulties (e.g., the Tinnitus Functional Index, Meikle et al., 2012; Henry et al., 2016) as one of the assessed subscales, none of them was designed to deal with the auditory processing issues undergone by tinnitus patients. In this work a new 25-item questionnaire, the Auditory Behavior Questionnaire (ABQ), is used to assess the auditory processing difficulties associated to hearing impaired subjects. This questionnaire can be useful to complement current measures of auditory processing deficits, like speech-in-noise tests, in tinnitus sufferers (Gilles et al., 2016).

In a first stage, the questionnaire was based on 114 items, including questions about the auditory functions that could be altered as a consequence of the APD. A pre-test pilot study helped to choose the correct extension of the questions, the resistance or rejection degree to some of them and the time needed for completion. This pilot study was led by a consulting panel, consisting of three ENT specialists with substantial experience with hearing impaired patients, one audiologist with direct experience with APD, and one psychologist expert in development of questionnaires.

In a second stage, the 114 initial items were reduced to 25 final items. The main criteria for choosing the 25 final items from these initial 114 were the descriptive analysis and the repetition frequency of each one. The opinion of patients was also taken into account, asking them to rank the items with which they felt more identified. The final 25 items were those that got a greater punctuation from patients. All items were related with the altered processing functions reported by the ASHA-American Speech-Language-Hearing Association (2005), as well as with the alteration of cognitive functions as attention, memory and auditory comprehension. Furthermore, a 25-item questionnaire should facilitate the inter-comparison with a well-established 25-item questionnaire for tinnitus, the THI. The original version in Spanish of the ABQ questionnaire is shown in Figure S1. For understanding purposes to non-Spanish readers, an English adaptation of the items (non-tested and non-validated) is provided in Figure S2. Factor analysis was applied for reliably grouping these items into 4 subscales.

### Scoring of the ABQ

Likewise as with THI, each patient response of the ABQ is rated as 0 (no), 2 (sometimes), or 4 (yes). Therefore, the total score of the ABQ, the sum of the individual responses, ranges between 0 and 100. Auditory processing handicap is then rated as slight (ABQ≤28), moderate (29≤ABQ≤58) or severe (ABQ≥59).

### Statistical analyses

Firstly, a factor analysis is applied for categorizing the 25 items into 4 subscales. Then, the internal consistency of each subscale and the full questionnaire is assessed by the Cronbach's alpha coefficient (α). α values greater than 0.7 are considered to provide acceptable internal consistency (Müller et al., 2016). Test-retest allowed checking the reliability of the questionnaire over the time by the intraclass correlation coefficient, *ICC*.

Age, ABQ score, AAT score (as calculated by Equation 1), and THI score (Spanish version) will be the statistical variables for this analysis. A descriptive analysis will be carried out for each of the subgroups defined in Table 1.

Finally, Spearman rank correlation analysis will be applied to paired variables for each subgroup. Spearman rank correlation will be used to identify and test the strength of relationships between these variables. Positive Spearman correlation coefficients (ρ) between *x* and *y* variables denote that both variables increase monotonically, and vice-versa, a negative correlation coefficient indicates that when *x* increases *y* decreases monotonically. The correlation between the variables is considered to be very weak for |ρ| ≤ 0.2, weak for 0.2 < |ρ| ≤ 0.4, moderate for 0.4 < |ρ| ≤ 0.6, strong for 0.6 < |ρ| ≤ 0.8 and very strong for |ρ| > 0.8. Omitting algebraic signs, when comparing different questionnaires for the same construct, ρ ≥ 0.4 denotes that both measure the same construct (convergent validity), whilst ρ < 0.4 signifies that both measure different aspects (discriminant validity) (Müller et al., 2016).

Data sets will be analyzed with MATLAB and R, with significance level *p* ≤ 0.05.

## Results

### Factor analysis

The 25 questions were aimed to identify those aspects of the auditory processing that can produce some kind of disablement related to the auditory processing capabilities, such as the attentional and memory capacity or selective attention, the auditory discrimination capability, the time aspects required for the correct comprehension of the sound message, the comprehension and integration of information, including discrimination and multisensory integration, the ability to structure thoughts and to coordinate auditory process with non-verbal auditory information, and the ability to assess space orientation features. A factor analysis was carried out to identify the underlying subscales.

**Figure 1:**
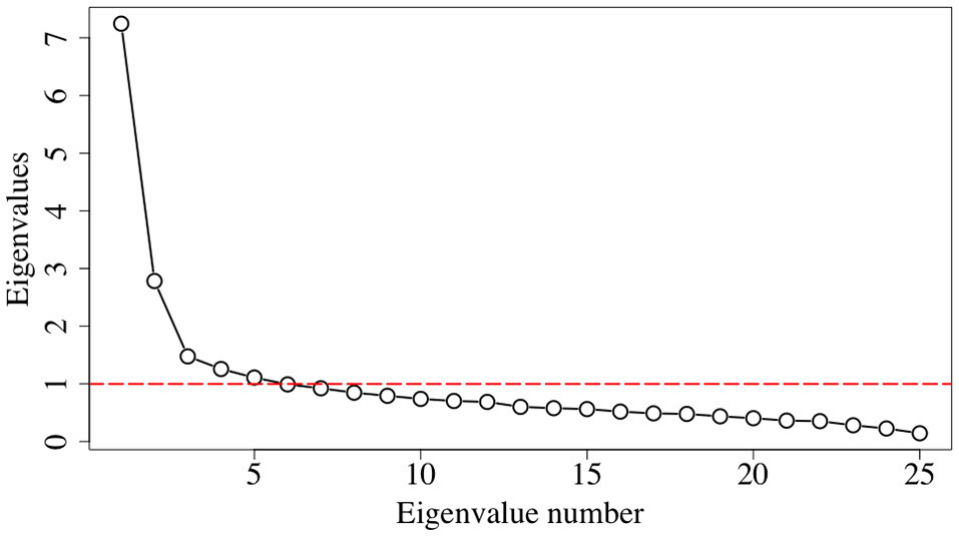
**Scree plot of the test correlation matrix**.

Table 2 summarizes the correlation values between the ABQ items, arranged in a way that stands out the similarities. It can be seen that there are a subset of items with higher correlation values, thus a pattern can be envisaged that allows discerning three or four significant groups of items. A scree plot, Figure 1, shows that there are 5 eigenvalues greater than 1. On the other hand, a parallel analysis suggests 3 as the number of factors to be retained in the analysis. A comparison between models with 3, 4, and 5 factors evidenced that the 5-factor model provided latent factors with only 2 items that, in turn, were closely related to items from other factors. In addition, some parameters (as the Root Mean Square Error of Approximation, RMSEA) proved a poor performance of the 3-factor model. Therefore, the 4-factor model based on principal axis factoring with oblique rotation was selected. This model gave a χ^2^ = 283.25, with 206 degrees of freedom, and a RMSEA = 0.061, what seems adequate to our case (Hutchinson and Olmos, 1998).

**Table 2:** **Correlation values between the questionnaire items ordered to show compact groups of values**.

	**1**	**2**	**7**	**5**	**9**	**6**	**12**	**10**	**11**	**3**	**4**	**14**	**16**	**18**	**19**	**17**	**20**	**21**	**22**	**23**	**8**	**13**	**24**	**15**	**25**
1	–																								
2	0.67	–																							
7	0.25	0.32	–																						
5	0.32	0.46	0.34	–																					
9	0.36	0.49	0.35	0.62	–																				
6	0.37	0.47	0.36	0.52	0.48	–																			
12	0.33	0.52	0.36	0.51	0.56	0.61	–																		
10	0.03	0.23	0.26	0.20	0.21	0.18	0.22	–																	
11	0.38	0.54	0.39	0.49	0.43	0.49	0.54	0.26	–																
3	0.34	0.55	0.29	0.41	0.31	0.40	0.39	0.25	0.53	–															
4	0.35	0.59	0.27	0.43	0.38	0.43	0.45	0.31	0.55	0.83	–														
14	0.25	0.42	0.28	0.36	0.38	0.38	0.45	0.24	0.38	0.39	0.40	–													
16	0.18	0.39	0.25	0.39	0.38	0.36	0.45	0.24	0.42	0.46	0.50	0.44	–												
18	0.12	0.19	0.30	0.15	0.23	0.13	0.18	0.14	0.17	0.19	0.12	0.07	0.14	–											
19	0.04	0.11	0.13	0.18	0.12	0.07	0.18	0.08	0.11	0.15	0.15	0.08	0.06	0.38	–										
17	0.05	0.13	0.25	0.04	0.08	0.20	0.24	0.12	0.12	0.17	0.13	0.11	0.09	0.35	0.26	–									
20	0.09	0.18	0.29	0.24	0.17	0.19	0.25	0.11	0.18	0.24	0.19	0.16	0.19	0.28	0.35	0.28	–								
21	0.03	0.09	0.16	0.14	0.13	0.15	0.15	0.10	0.14	0.18	0.19	0.11	0.21	0.33	0.30	0.33	0.40	–							
22	0.06	0.12	0.27	0.17	0.17	0.19	0.26	0.15	0.11	0.14	0.20	0.16	0.15	0.27	0.36	0.32	0.40	0.46	–						
23	0.10	0.07	0.28	0.10	0.15	0.11	0.12	0.21	0.17	0.10	0.06	0.12	0.04	0.34	0.26	0.28	0.21	0.20	0.29	–					
8	0.20	0.25	0.37	0.18	0.18	0.09	0.15	0.23	0.28	0.24	0.19	0.17	0.17	0.31	0.19	0.17	0.15	0.16	0.18	0.39	–				
13	0.20	0.21	0.31	0.17	0.18	0.17	0.20	0.20	0.20	0.17	0.19	0.20	0.17	0.23	0.18	0.25	0.18	0.14	0.21	0.41	0.53	–			
24	0.14	0.24	0.31	0.27	0.23	0.16	0.25	0.20	0.20	0.20	0.20	0.30	0.17	0.19	0.28	0.16	0.35	0.19	0.24	0.31	0.18	0.25	–		
15	0.19	0.18	0.28	0.11	0.16	0.22	0.20	0.11	0.22	0.26	0.24	0.23	0.29	0.32	0.21	0.30	0.32	0.39	0.22	0.24	0.28	0.24	0.22	–	
25	0.07	0.20	0.25	0.13	0.13	0.22	0.15	0.17	0.19	0.24	0.24	0.19	0.24	0.27	0.15	0.19	0.19	0.24	0.20	0.26	0.29	0.17	0.23	0.40	–

Table 3 summarizes the loadings of the items as a function of the factors. The 4 subscales resulting from this analysis have then been defined as:

- Auditory Discrimination (AD): consists of 8 items and is expected to assess attention, memory, auditory discrimination, and the time aspects required for the correct comprehension of the sound message.
- Multisensorial Integration (MI): consists of 9 items supposed to evaluate comprehension and integration of information, as well as discrimination and multisensorial integration.
- Concentration Capacity (CC): consists of 5 items expected to pick up the selective attention and difficulties to concentrate in sound environments.
- Understanding Capacity (UC): consists of 3 items that would allow measuring the ability to understand speech in noisy and reverberant environments.

**Table 3:** **Loadings of the items for the resulting factors**.

**ID**	**AD**	**MI**	**CC**	**UC**
9	0.86			
12	0.85			
5	0.8			
6	0.75			
2	0.57			0.27
11	0.51			0.24
1	0.46			
14	0.45			
21		0.77		
20		0.65		
22		0.65		
19		0.54		
17		0.48		
18		0.41	0.25	
15		0.41		
24		0.27		
25		0.24	0.23	
8			0.89	
13			0.71	
23			0.59	
7	0.34		0.35	
10			0.23	
4				0.82
3				0.81
16	0.34			0.34

**Figure 2:**
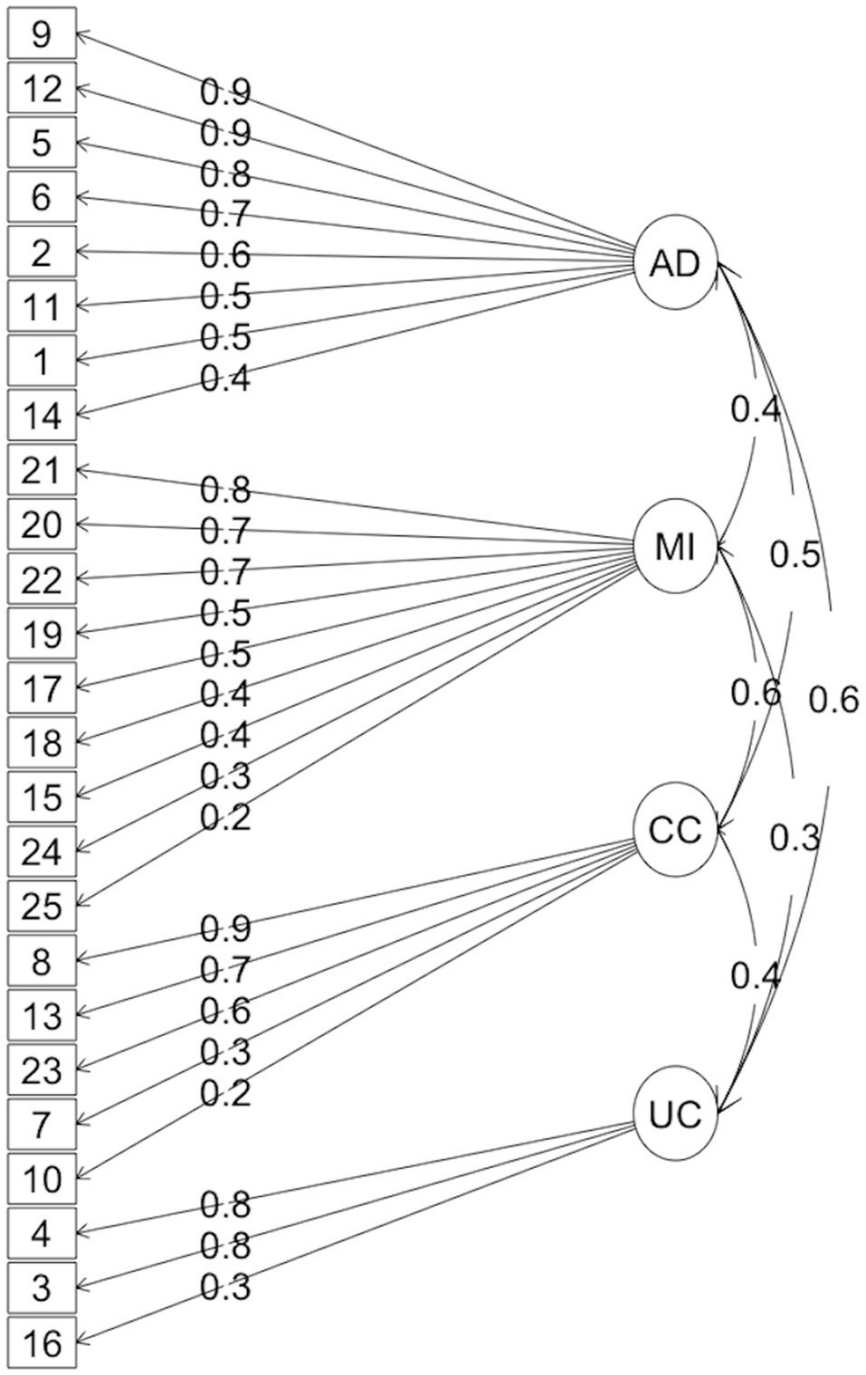
**Diagram of the resulting model from the factor analysis**.

The resulting model is shown in Figure 2. The English version of the final questionnaire (originally in Spanish), defined according to this model, is shown in Figure S2. Notice that this English version consists of a translation by the authors and has not been rigorously tested nor validated (Müller et al., 2016). Users interested in using this version should request permission to use to the first author (idiges8@gmail.com).

### Reliability

Table 4 summarizes the results of the reliability tests. Third column shows the results for the internal consistency reliability tests that turns out to have an α above 0.75 for all the subscales, except for “Concentration Capacity” (α = 0.69) which is yet acceptable.

**Table 4:** **Initial and test-retest Cronbach's alpha for each subscale of the ABQ**.

**Subscale**	**No. of Items**	**α_initial_(*N* = 310)**	***ICC*_test−retest_(*N* = 35)**
Overall	25	0.89	0.91
AD	8	0.87	0.77
MI	9	0.78	0.88
CC	5	0.69	0.83
UC	3	0.81	0.75

Fourth column of Table 4 shows the values of the *ICC* resulting of applying the test-retest to 35 patients. As it can be seen test-retest reliability evidences a good consistency for all the subscales.

### Descriptive analysis of variables

**Figure 3:**
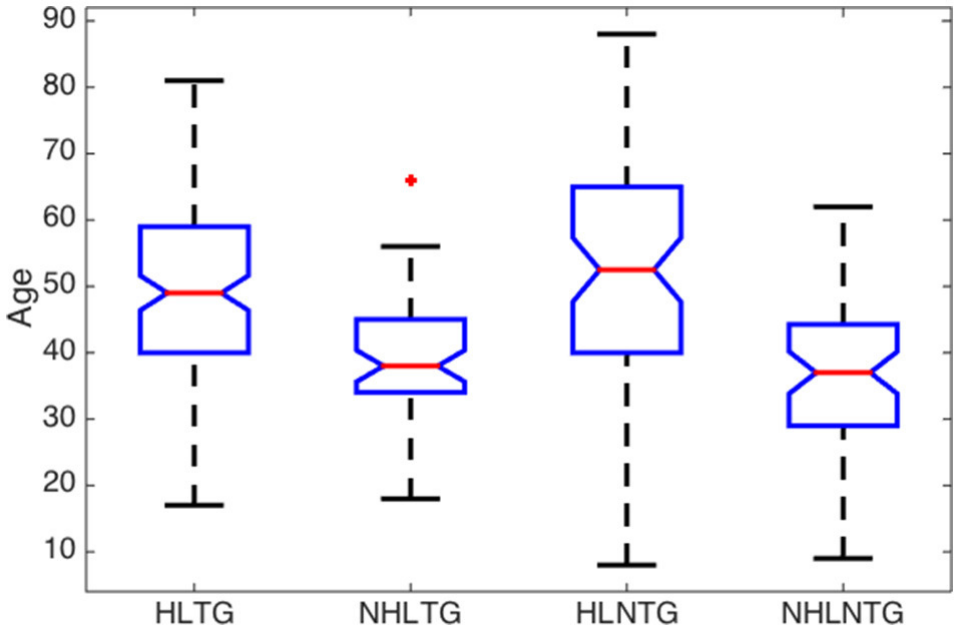
**Boxplot of descriptive analysis of age by subgroups**.

**Figure 4:**
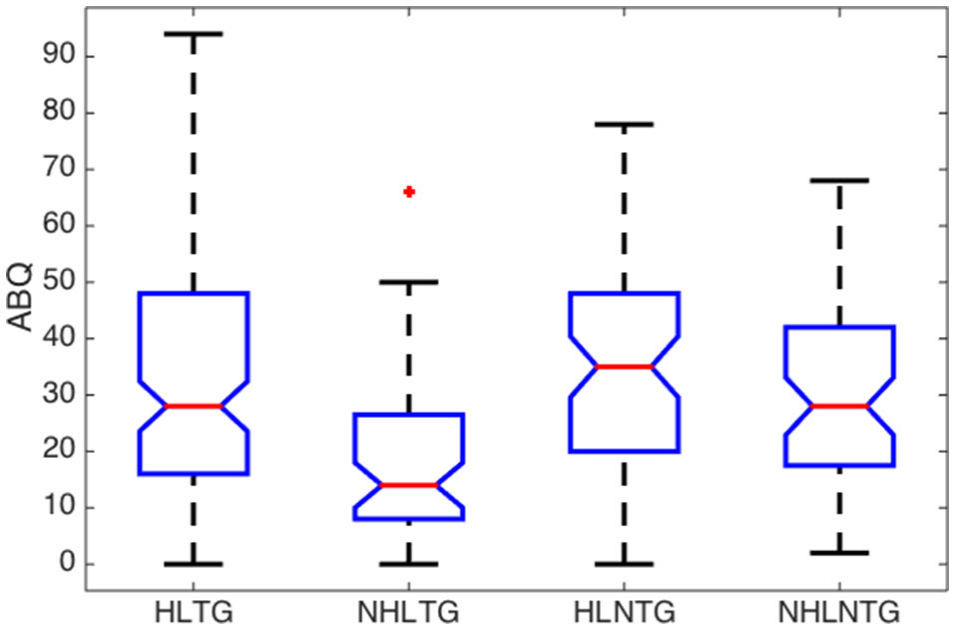
**Boxplot of descriptive analysis of ABQ by subgroups**.

**Figure 5:**
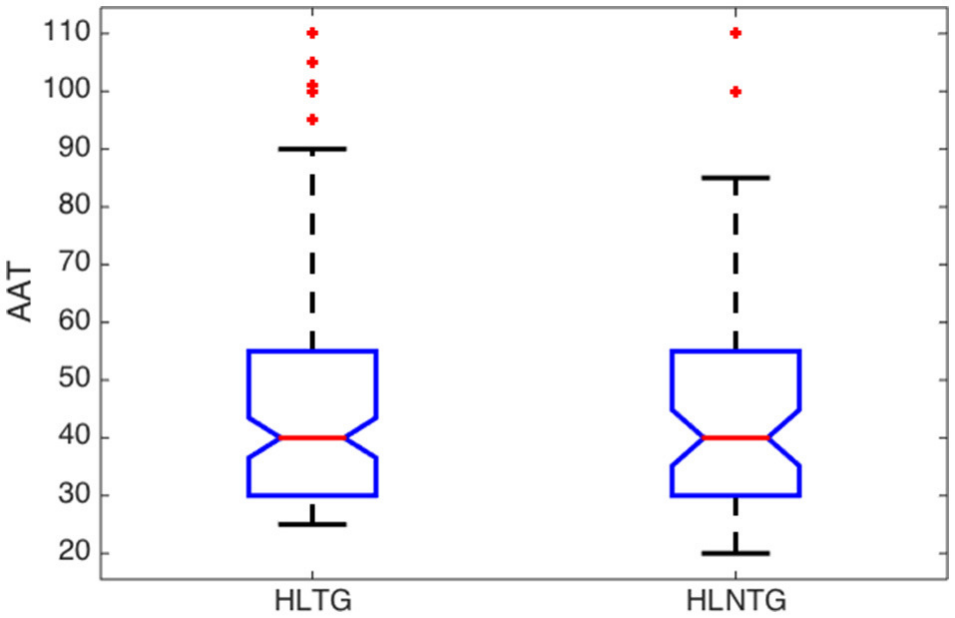
**Boxplot of descriptive analysis of AAT by subgroups**.

**Figure 6:**
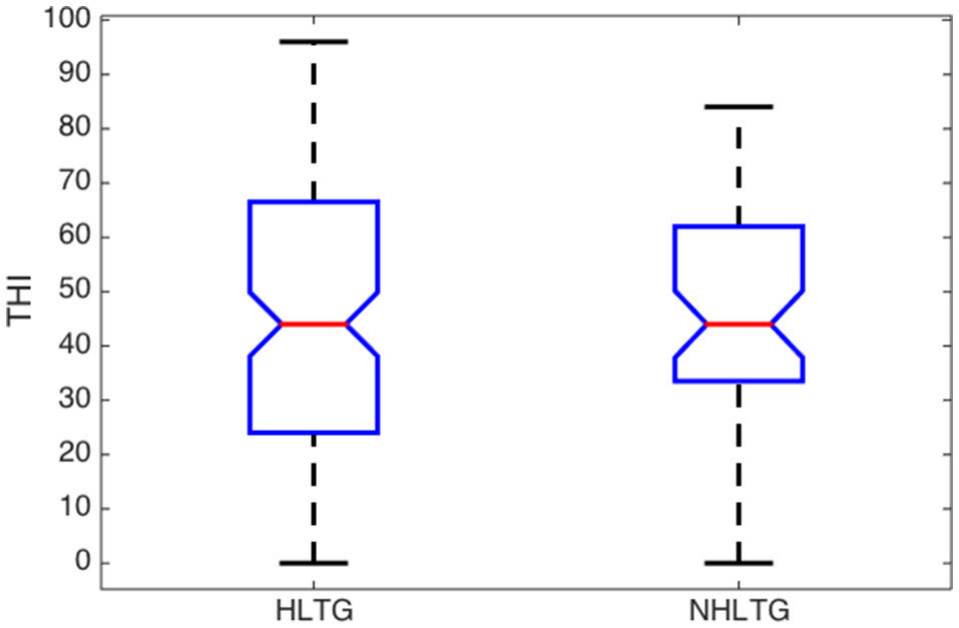
**Boxplot of descriptive analysis of THI by subgroups**.

Some relevant statistical parameters of the variables (Age, ABQ, AAT, and THI) for each subgroup are summarized in Table 5 and Figures 3–6.

**Table 5:** **Descriptive analysis for the variables of the four subgroups**.

	**Age**				**ABQ**				**AAT**		**THI**	
	**HLTG**	**HLNTG**	**NHLTG**	**NHLNTG**	**HLTG**	**HLNTG**	**NHLTG**	**NHLNTG**	**HLTG**	**HLNTG**	**HLTG**	**NHLTG**
*N*	129	66	53	57	129	66	53	57	129	66	129	53
Median	49	52.5	38	37	28	35	14	28	40	40	44	44
*SD*	12.7	15.6	9.2	11.0	19.6	19.5	13.8	16.7	22.1	22.0	25.4	22.1
Skewness	0.081	−0.23	0.49	0.013	0.44	0.17	1.032	0.451	1.29	1.087	0.11	−0.11

A significant difference in age was found between the median of patients with or without HL, with a confidence level of 95%, regardless they suffer from tinnitus or not, Figure 3. The median ages of patients in NHLTG and NHLNTG are 11 and 15.5 years lower than HLTG and HLNTG, respectively. Both NHLTG and NHLNTG also have a smaller variability as compared to the HLTG and HLNTG. Thus, in average, patients without HL are younger than patients with HL.

Regarding the ABQ, and considering the median values for all the subgroups, patients exhibit a moderate processing disorder (29<ABQ≤58) in the HLNTG (thus, with HL and without tinnitus), and a slight processing disorder (ABQ<28) in the HLTG, NHLTG, and NHLNTG. The skewness of the ABQ for the different subgroups is much greater for the NHLTG than for the rest of the subgroups, so that the relative amount of cases with high ABQ scores for this subgroup is greater than in the other subgroups. Furthermore, AAT distribution is more asymmetric than the rest of variables. Also, AAT and THI values of patients in the different subgroups do not show significant differences, Figures 5, 6.

### Rank correlations

Table 6 summarizes the Spearman rank correlation coefficients between paired statistical variables. As it can be seen, AAT exhibits a strong monotonic increasing relationship with age for patients in the HLNTG subgroup, and a weak relationship in the HLTG subgroup. Thus, HL seems to increase strongly with age for patients without tinnitus, and weakly for patients with tinnitus. ABQ shows a weak monotonically decreasing relationship with age for patients in the NHLTG subgroup. Furthermore, the relationship between ABQ and THI is moderate for the HLTG subgroup, and weak for the NHLTG subgroup. Hence, ABQ increases moderately or weakly with THI in patients without or with HL, respectively. The interrelationship of ABQ with AAT is weak for both HLTG and HLNTG subgroups. All the other paired variables reveal a very weak interrelationship (|ρ| < 0.2).

**Table 6:** **Spearman rank correlation coefficients between variables for each subgroup**.

	**Age**	**ABQ**	**AAT**	**THI**
ABQ	0.13 (HLTG)			
	0.13 (HLNTG)			
	−0.31 (NHLTG)	1		
	0.06 (NHLNTG)			
AAT	0.34 (HLTG)	0.31 (HLTG)	1	
	0.63 (HLNTG)	0.21 (HLNTG)		
THI	−0.06 (HLTG)	0.44 (HLTG)	−0.02 (HLTG)	1
	−0.17 (NHLTG)	0.34 (NHLTG)		

**Figure 7:**
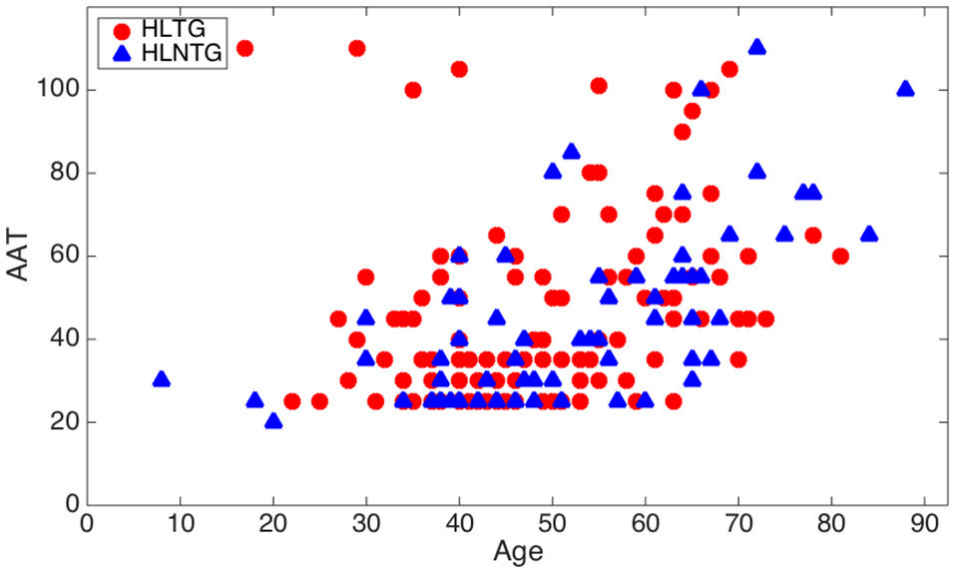
**Scatter plot of AAT vs. age for the HLTG and HLNTG subgroups**.

**Figure 8:**
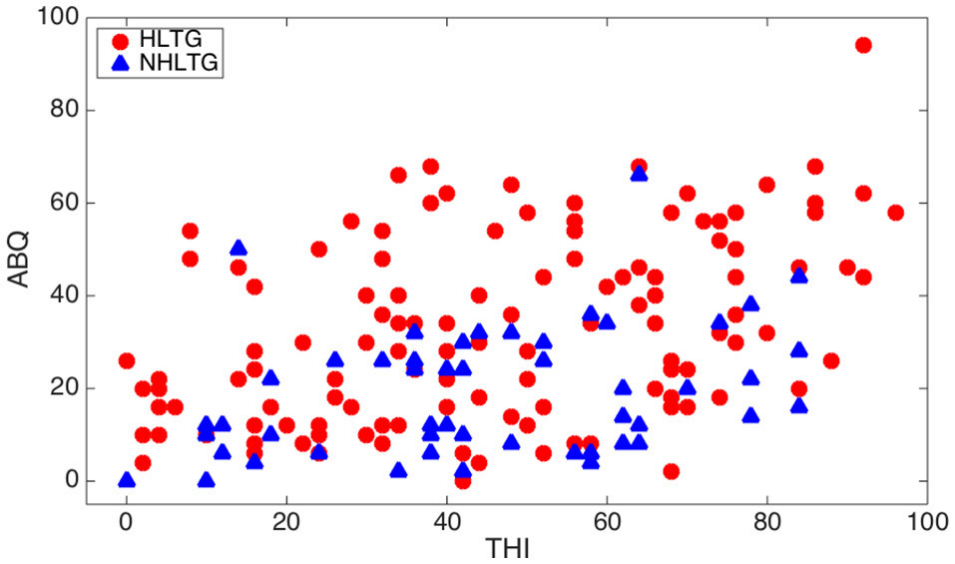
**Scatter plot of ABQ vs. THI for the HLTG and NHLTG subgroups**.

Table 7 shows the expected inter-relationships between the analyzed hearing impairments as well as their variation with age. The usually reported increasing HL with age is confirmed in our results of Table 6. Furthermore, the rank correlation coefficient is almost double for patients without tinnitus (HLNTG) in comparison to patients with tinnitus (HLTG). For illustrating better some of these inter-relationships, Figures 7, 8 show the scatter plots of AAT vs. Age, for the HLTG and HLNTG subgroups, and ABQ vs. THI, for the HLTG and NHLTG subgroups, respectively.

**Table 7:** **Expected inter-relationship between Age, HL, Tinnitus, and APD**.

	**Age**	**APD**	**HL**	**Tinnitus**
APD	⇑		⇑	⇑
HL	⇑	≈		≈
Tinnitus	⇑	≈	⇑	

## Discussion

The descriptive analysis of the statistical variables has provided remarkable results. Firstly, it was found that the average age of patients without HL is significantly lower that the corresponding average age of patients with HL, see Figure 3. This is consistent with the fact that younger people begins to experience auditory troubles even though HL has not been developed yet. Many authors attribute these troubles to noise overexposure (Emmerich et al., 2002; Gilles et al., 2016). According to Liberman and Liberman (2015), diffuse loss of internal hair cells (IHC), or the auditory nerve fibers they innervate, has to exceed 80–90% before auditory thresholds increase significantly. Secondly, for patients with tinnitus, the average ABQ score is significantly lower (half) without HL than with HL (see Table 5). In other words, HL seems to weaken the auditory processing capabilities of subjects with tinnitus. Hyvärinen et al. (2016) found that tinnitus may degrade auditory localization ability, although this effect can also be due to the associated levels of HL. And thirdly, we have not found differences between the average THI score in patients with or without HL (see Figure 6). In principle, this could contradict the finding that subjective discomfort seems to be higher in tinnitus patients with HL than in those without HL (Ganz Sanchez et al., 2005; Savastano, 2008). Nevertheless, if we take into consideration that, in our patients cohort, the average age of subjects in the NHLTG subgroup is 11 years lower than those in the HLTG subgroup, this result reinforce the idea that tinnitus in young people can be triggered by damage in the IHC-auditory nerve synapses (Liberman and Liberman, 2015).

The prevalence of APD is expected to increase with age (Skarzynski et al., 2015). Our results provided a weak monotonic increasing dependence of ABQ with age for subjects with HL (HLTG and HLNTG), see Table 6. For patients with normal hearing, this dependence is decreasing for patients with tinnitus (NHLTG) and practically plane for patients without tinnitus (NHLNTG). Thus, the expected increasing dependence of ABQ with age is not confirmed in patients with normal hearing.

APD is also expected to increase with HL (George et al., 2007). However, our results provided a weak correlation of ABQ with AAT, regardless the patients are suffering of tinnitus or not, see Table 6.

The increasing interdependency between APD and tinnitus has been reported by some authors. Newman et al. (1994) investigated the relationship between psychoacoustic judgements, speech understanding ability and self-perceived handicap in tinnitus and hearing impaired subjects. Hyvärinen et al. (2016) reported that tinnitus may degrade auditory localization ability, although this effect is, for the most part, due to the associated levels of HL. Gilles et al. (2016) showed that young people with noise induced tinnitus, but normal hearing thresholds, proved impaired speech-in-noise performance. Jain and Sahoo (2014) found that tinnitus has an effect on certain aspects of auditory processing like temporal resolution, speech perception in noise and frequency discrimination in individuals with normal hearing. Our results confirm a monotonically increasing inter-relationship between ABQ and THI, moderate for patients with concomitant HL (HLTG), and weak for patients with normal hearing (NHLTG).

We have not found significant correlation of THI score with age, which does not contradict the generalized idea that the incidence of tinnitus increases with aging, since THI measures negative reactions of tinnitus, not its prevalence.

Most patients of tinnitus have a related HL, attributable to aging, noise exposure, or chronic otitis media (Holm et al., 2005). Although, HL is an important risk factor for tinnitus, this can occur independently from broad increase of hearing thresholds. Normal hearing thresholds can also be accompanied by impaired function of efferent fibers that project from the brainstem to the cochlea (Schaette and McAlpine, 2011). We have not found a significant correlation factor between THI and AAT, see Table 6. Therefore, our results do not support the generalized belief that tinnitus increases with HL. Again, this result reinforces the model of tinnitus triggered by cochlear synaptopathy, which can occur due to noise overexposure (Liberman and Liberman, 2015; Gilles et al., 2016).

The subjective discomfort has been reported to be higher in tinnitus patients with HL than in those without HL (Ganz Sanchez et al., 2005; Savastano, 2008). However, we have found the same average THI in patients with HL (HLTG) and normal hearing (NHLTG), see Table 5.

### Limitations

Since audiological assessment of subjects was obtained at a single point of time, the study presented here is cross-sectional in itself. Thus, although the results have been interpreted as an estimation of co-occurring hearing impairments in a cohort, they should not be given a prospective significance. The current study, as applied in this work, has been used to analyse comorbidities between hearing impairments, but cannot discriminate between causes and effects. It is also worth mentioning that the above discussed results and interpretations have the known limitations of behavior science studies. Namely, correlational analysis is able to assess the direction and strength of inter-relationships between paired variables but does not provide causal links between them (Stangor, 2011). Thus, the correlational analysis applied in this work has demonstrated that some of the variables are associated, as discussed above, but this relationship could be due to another external variable. Furthermore, although factor analysis results suggest the existence of four latent variables, the need of using oblique rotation, together with the value of the loadings of some of the items, suggests the existence of interactions between them that would require further in-depth studies. It should be considered that the analysis reported here was based on descriptive analysis which has not controlled for the effects of other explanatory and confounding variables such as age, sex, AAT, THI, or type of work experience, and environmental (noise levels) or biological factors. Also, we are currently collecting relevant data, firstly, to carry out statistical diagnostic on ABQ in predicting HL, and secondly, to allow control for other variables in the statistical analysis.

## Conclusions

An inter-relationship study between hearing loss, tinnitus and auditory processing disorder in 305 patients attending an audiological clinic has been carried out in this work. Such audiological disorders have been measured by the average auditory threshold, tinnitus handicap inventory and auditory behavior questionnaire, respectively. The results of this study have confirmed the expected monotonically increasing dependency of auditory behavior questionnaire score and average auditory threshold with age, as well as auditory behavior questionnaire score with average auditory threshold and tinnitus handicap inventory score. However our results, unlike those previously reported by others, show that tinnitus handicap inventory score does not increase with either age or average auditory threshold.

## Ethics statement

The study presented in this manuscript contains a post-analysis of tests and questionnaires from a dataset that did not require approval from a medical ethical board. All the patients attending an audiological clinic (ACURE) were subjected to standard audiological tests and asked to fill the questionnaires. Thus, the analyses we include in this manuscript resulted from a post-processing of these data, paying special attention to keep all patients de-identified. Anyway, the study would be approved by an ethical committee if it had been submitted.

## Author contributions

ID designed the ABQ. FS and PC analyzed the data. PC and FS drafted the initial and final versions of the manuscript.

### Conflict of interest statement

The authors declare that the research was conducted in the absence of any commercial or financial relationships that could be construed as a potential conflict of interest.
