# Supplementary material for: Assessing Auditory Processing Deficits in Tinnitus and Hearing Impaired Patients with the Auditory Behavior Questionnaire
Source: Front Neurosci. 2017 Apr 6;11:187. doi: 10.3389/fnins.2017.00187 (PMC5382167; doi:10.3389/fnins.2017.00187)
Supplement: Supplementary file 1 [file Image1.PDF]

CUESTIONARIO DE COMPORTAMIENTO AUDITIVO (CCA)

Diges I, Herráiz C.

Instrucciones: Ponga una cruz en la respuesta que mejor describa su situación.
Por favor, no deje ninguna respuesta sin contestar.

		Si	A veces	No
1C	En ambiente silencioso, ¿tiene problemas para entender bien una conversación de persona a persona?			
2C	En ambiente silencioso, ¿tiene problemas para entender bien una conversación con un grupo de personas?			
3C	En ambiente con ruido de fondo, ¿tiene problemas para entender bien una conversación de persona a persona?			
4C	En ambiente con ruido de fondo, ¿tiene problemas para entender bien una conversación con un grupo de personas?			
1R	Si le hablan rápido ¿entiende peor?			
2R	En ocasiones , ¿entiende una palabra por otra?			
3R	¿Tiene dificultad para seguir instrucciones en varios pasos?			
4R.	¿Tiene dificultad para concentrarse?			
5R.	¿Le cuesta entender las palabras por separado cuando le hablan rápido?			
6R.	¿Le resultan molestos algunos sonidos del entorno?			
7R.	¿Repite frecuentemente, qué y éh, durante las conversaciones?			
8R.	¿Confunde palabras o consonantes que suenan parecidas?			
9R	¿Se distrae con facilidad?			
10R	¿Tiene dificultad para localizar los sonidos?			
11R	¿Tiene dificultad para recordar nombres y lugares?			
12R	En salas con reverberación (eco) ¿le cuesta entender?			
1E.	¿Cuándo habla se confunde o invierte las palabras?			
2E.	¿Tiene dificultad para comprender lo que lee?			
3E	¿Tiene dificultad al leer, especialmente en voz alta?			
4E	¿Tiene dificultad para aprender un idioma o repetir una palabra difícil?			
5E.	¿Le cuesta encontrar la palabra adecuada para expresarse correctamente?			
6E	¿Tiene dificultad para hablar con fluidez y velocidad adecuada?			
1AE.	¿Tiene dificultad para organizarse o planificar tareas?			
2AE	¿Tiene dificultad para seguir el ritmo de la música?			
3AE	¿Tiene dificultad para orientarse?			

Total _________

© 2009. Todos los derechos reservados

Figure S1. Original version (in Spanish) of the ABQ.
