# Supplementary material for: Assessing Auditory Processing Deficits in Tinnitus and Hearing Impaired Patients with the Auditory Behavior Questionnaire
Source: Front Neurosci. 2017 Apr 6;11:187. doi: 10.3389/fnins.2017.00187 (PMC5382167; doi:10.3389/fnins.2017.00187)
Supplement: Supplementary file 2 [file Image2.PDF]

AUDITORY BEHAVIOUR QUESTIONNAIRE

**Name:**	**Date:**

Please read carefully the following questions and answer by drawing an X in the corresponding box

**AD. Auditory Discrimination**			
	**Yes**	**Sometimes**	**No**
1. Do you have trouble to understand other person in a quiet environment?	☐	☐	☐
2. Do you have trouble to understand a group of people in a quiet environment?	☐	☐	☐
5. Does your listening become worse if the person you are talking to speaks faster?	☐	☐	☐
6. Do you sometime confound a word for other?	☐	☐	☐
9. Do you have difficulties to understand separate words if if the person you are talking to speaks fast?	☐	☐	☐
11. Do you often answer “what” or “eh” when talking with others?	☐	☐	☐
12. Do you confuse words that sound similar when talking to others?	☐	☐	☐
14. Do you have difficulties to locate sound?	☐	☐	☐
**MI. Multisensorial Integration**			
	**Yes**	**Sometimes**	**No**
15. Do you have difficulties to remember names and locations?	☐	☐	☐
17. Do you confuse or invert words when speaking?	☐	☐	☐
18. Do you have difficulties in comprehending what you read?	☐	☐	☐
19. Do you have reading difficulties, especially out loud?	☐	☐	☐
20. Do you have difficulties to learn a foreign language or to repeat a long word?	☐	☐	☐
21. Do you have difficulties to find the correct word in a spoken sentence?	☐	☐	☐
22. Do you have difficulties to speak fluently?	☐	☐	☐
24. Do you have difficulties to follow a musical rhythm?	☐	☐	☐
25. Do you have difficulties in orientating yourself?	☐	☐	☐
**CC. Concentration Capacity**			
	**Yes**	**Sometimes**	**No**
7. Do you have difficulties to follow step-by-step instructions?	☐	☐	☐
8. Do you have difficulties to focus attention?	☐	☐	☐
10. Are you annoyed by some environmental sounds?	☐	☐	☐
13. Do you often become absent-minded?	☐	☐	☐
23. Do you have difficulties when organizing and planning tasks?	☐	☐	☐
**UC. Understanding Capacity**			
	**Yes**	**Sometimes**	**No**
3. Do you have trouble to understand other person in a noisy environment?	☐	☐	☐
4. Do you have trouble to understand a group of people in a noisy environment?	☐	☐	☐
16. Do you have difficulties for understanding in reverberant environments?	☐	☐	☐

		**Slight (ABQ≤28)**	☐
**Total Score___________**	**Auditory Processing Disorder is**	**Moderate (30≤ABQ≤58)**	☐
		**Severe (ABQ≥60)**	☐

Figure S2. Authors English adaptation (untested and non-validated) of the ABQ.
